# A Benchmark Study of Graph Models for Molecular Acute Toxicity Prediction

**DOI:** 10.3390/ijms241511966

**Published:** 2023-07-26

**Authors:** Rajas Ketkar, Yue Liu, Hengji Wang, Hao Tian

**Affiliations:** 1 Yale College, Yale University, New Haven, CT 06520, USA; 2 Department of Chemistry, University of Washington, Seattle, WA 98195, USA; 3 Department of Physics, University of Washington, Seattle, WA 98195, USA; 4 Department of Chemistry, Southern Methodist University, Dallas, TX 75275, USA

**Keywords:** graph neural network, acute toxicity, attentive FP, machine learning

## Abstract

With the wide usage of organic compounds, the assessment of their acute toxicity has drawn great attention to reduce animal testing and human labor. The development of graph models provides new opportunities for acute toxicity prediction. In this study, five graph models (message-passing neural network, graph convolution network, graph attention network, path-augmented graph transformer network, and Attentive FP) were applied on four toxicity tasks (fish, *Daphnia magna*, *Tetrahymena pyriformis*, and *Vibrio fischeri*). With the lowest prediction error, Attentive FP was reported to have the best performance in all four tasks. Moreover, the attention weights of the Attentive FP model helped to construct atomic heatmaps and provide good explainability.

## 1. Introduction

The growing utilization of various chemicals, including medicines, pesticides, and chemical fertilizers, has sparked increasing interest due to their considerable toxicity [1]. Apart from criticism for animal testing, assessing the toxicity and risk of chemicals is a complex task that often requires significant time and effort [2]. Therefore, the research for chemical toxicity assessment is crucial in the advancement of alternative methods to animal testing and will support the development of predictive modeling [3].

Many drugs also fail clinical trials due to their unmanageable toxicity, which is a significant cause of the high failure rate in clinical drug development. To alleviate this problem in an already long and costly process, it would be ideal to gauge toxicity directly from the molecular structure [4]. However, this is a challenging task for humans because chemical systems are complex and such properties are not always immediately evident from molecular substructures. In fact, determining effective methods to represent molecules for toxicity prediction remains challenging, though many exist, including theoretical, empirical, and semi-empirical representations. Descriptors representing a molecular structure may be divided into graph-based and geometry-based types. Graph-based representations primarily describe the connectivity between atoms, while geometry-based representations include information about the molecule’s physical geometry, including bond lengths and angles [5].

Traditionally, many quantitative structure–toxicity relationship (QSTR) models have been introduced as a means to evaluate and predict chemical risk, relying solely on molecular structure information [6,7,8]. The molecular structure is featurized by physical and chemical properties such as size, covalent bonds, and polarity, and it can be effectively characterized through quantitative molecular descriptors. Among the most common descriptors for toxicity prediction are Molecular Access System keys (MACCS Keys), PubChem Substructure Fingerprints, Klekotha–Roth, and Extended-Connectivity Fingerprints (ECFPs) [9]. However, these models require predefined features and molecular descriptors, and a lack of exhaustiveness would lead to poor performance. With more than 5000 molecular descriptors available for characterizing molecules, it is not always clear how to select the most appropriate representation while avoiding selection bias. In place of such general descriptors, there has been growing interest in alternative data-driven representations via deep learning [5,10,11].

With the development of molecular graph representations and deep learning, graph neural networks (GNNs) are one of the leading research areas for molecular property prediction. In a molecular graph, atoms are treated as vertices and bonds are treated as edges. By functioning directly on molecular graphs, GNNs provide a more accurate description of the chemical structure with connectivity information and can allow for more relevant representations of molecules without having to consider many-body interactions or complex electronic structures [5,12,13]. Starting from message-passing neural networks (MPNNs) [14], many variations have been developed with higher complexity or additional attention mechanisms, such as the graph convolution network (GCN), graph attention network (GAT), path-augmented graph transformer network (PAGTN) [15], and Attentive FP model [5]. These architectures employ various techniques to overcome the complexity and irregularity of molecular graphs.

In recent years, deep learning has grown popular for toxicity prediction due to its successful performance and because it avoids a challenging feature selection process. For example, Mayr et al.’s DeepTox pipeline proved effective on the Tox21 data challenge and outperformed shallower machine learning methods [16]. The choice of molecular representation has also shown to be important in this domain, and graphs have been shown to be useful in encoding a molecular structure, as vertices can represent atoms and edges can represent connections between atoms [17]. In 2017, Xu et al., introduced molecular graph-encoding convolutional neural networks for acute oral toxicity (AOT) prediction via their deepAOT model [10]. This model uses graph convolutions to represent molecules, removing the need to calculate fingerprints and allowing the algorithm to learn more directly from the data. Wu and Wei presented another topology-based molecular representation in 2018 that paired element-specific persistent homology with multitask deep learning and proved successful on four benchmark toxicity datasets [18].

In this study, the performance of five graph models (MPNN, GCN, GAT, PAGTN, and Attentive FP) was evaluated on four acute toxicity tasks (fish, *Daphnia magna*, *Tetrahymena pyriformis*, and *Vibrio fischeri*). With rigorous training and testing, Attentive FP was identified as the best-performing model with low prediction error. Furthermore, the attention weights from the trained Attentive FP model can help visualize atom importance with a molecular heatmap. This interpretability is especially important in molecular property prediction for understanding the model’s internal workings and gaining new scientific insights [19].

## 2. Results

### 2.1. Graph Models Training

**Figure 1:**
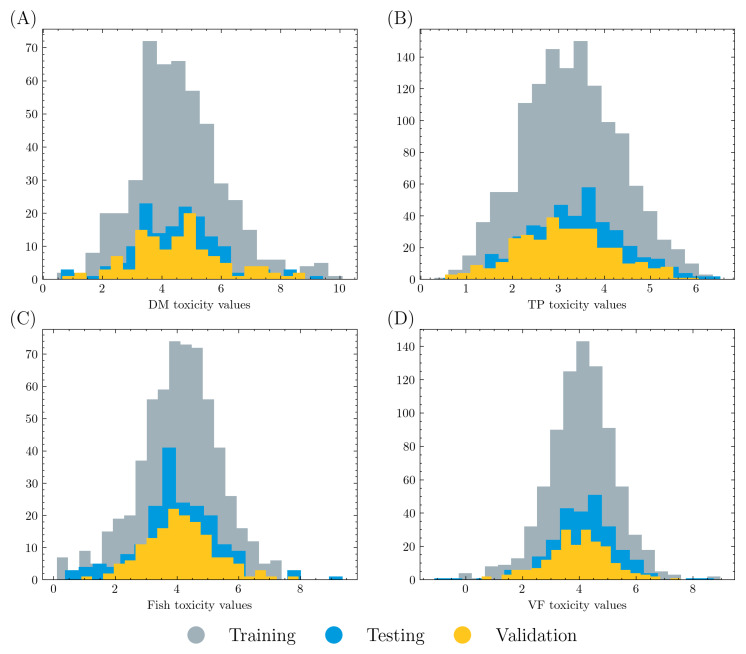
Count and value distribution in (**A**) DM, (**B**) TP, (**C**) fish, and (**D**) VF acute toxicity tasks. Training, validation, and testing data account for 64%, 16%, and 20% of the overall dataset, respectively. All three datasets in each task have similar distribution.

The overall dataset was randomly split into a training set (80%) and a test set (20%). Five-fold cross validation was applied on the training set, where 80% of the training data were used to train a graph model and 20% of the data were used for validation in each training process. Therefore, training, validation, and testing data account for 64%, 16%, and 20% of the overall dataset, respectively. The data distributions are displayed in Figure 1. It is shown that all three datasets have similar distributions in each acute toxicity task.

The parameters being fine-tuned in the graph models are listed in Table 1. The learning rate was fixed as 0.001 in all graph models. The batch size and dropout rate parameters were tuned in all models. The number of GNN layers was tuned for the PAGTN and Attentive FP models with potential values in 1, 2, and 4. The number of graph convolution layers was tuned for the GCN and GAT models from two layers of 64, 128, and 256 units. The MPNN model tuned a similar parameter of the number of units in the hidden layer. It also tuned the number of atom output features with 15, 30 and 60. Similarly, the PAGTN model tuned the size for the hidden node features with 64, 128, and 256. The selected parameters can be classified into sample size (batch size), regularization (dropout), and complexity (number of GNN layers, number of graph convolution layers, number of units, number of atom output features, and size for the hidden node features). The full combination of all the parameters was tested.

### 2.2. Superior Performance of Attentive FP

The mean MSE, MAPE, and R^2^ metrics were calculated under each parameter setting. The metric calculations are detailed in Equations (4)–(6). The model with the lower MSE and MAPE and higher R^2^ was considered to have better performance. The best model setting on the training set was taken and applied in the test set. The model was trained in five independent runs, and the mean and standard deviation are calculated and summarized in Table 2. In all four tasks, Attentive FP performed the best among the five graph models. Specifically, in the VF and TP tasks, Attentive FP was significantly better than the other models with a 12.3% and 13.3% decrease in the MSE as compared to the second-best GCN model, respectively. The GCN model was the second-best model with 3rd place in the DM task and 2nd place in the other three tasks. The other three models have varying performance in different tasks. For example, the GAT model was ranked 2nd in the DM task but last in the VF task. At the task level, the graph models had the best performance on the TP task and similar performance on the VF and fish tasks, with DM being the worst.

To further validate that graph models are truly reflective of a structure–activity relationship, a y-randomization test was performed for the Attentive FP and GCN models. The toxicity values on the training set were randomly shuffled and used for model training under the same best-performing hyperparameter sets. For the Attentive FP model, the R2 values were 0.0142, 0.0048, 0.0780, and 0.0126 for the VF, DM, TP, and fish tasks. For the GCN models, the corresponding values were 0.0591, 0.0568, 0.0624, and 0.0007.

**Figure 2:**
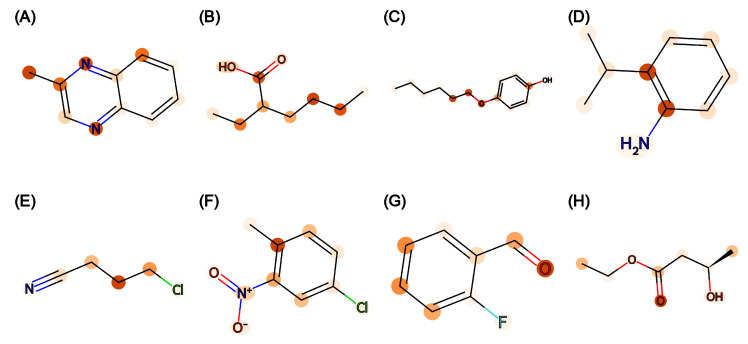
Molecular heatmap with attention weights of Attentive FP trained for the TP task. (**A**–**H**) are eight randomly selected molecules in the test set of TP task. In each molecule, atomic bonds are represented in black, blue, red, or green lines according to atom types. The atom with higher weight has darker color.

The attention weights on the attention layer of Attentive FP were extracted to produce molecular heatmaps. Figure 2 displays the heatmap of eight molecules with a low prediction error (<0.01) from the trained Attentive FP model on the TP task. It is shown that many carbon and nitrogen atoms on aromatic rings were assigned a high weight. While other graph models do not have weights at the atomic level, the attention weights and heatmap from the Attentive FP model can be used to explain model prediction against single prediction and can be extended for rational design.

## 3. Discussion

**Figure 3:**
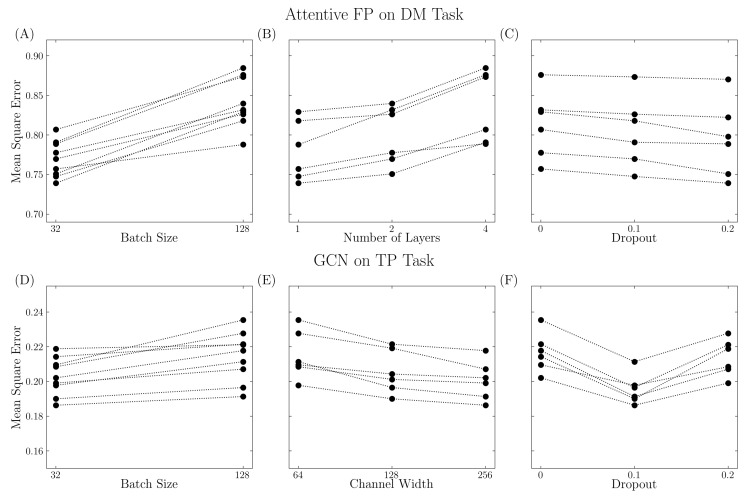
Various model performance with different parameter settings. The performance was quantified using mean square error. (**A**–**C**) Attentive FP model trained on DM task. (**D**–**F**) GCN model trained on TP task. Three factors were considered such as sample size (batch size parameter), model complexity (number of layers for Attentive FP model and channel width for GCN model), and regularization (dropout parameter).

Appropriate parameters are needed to reach the best performance. As shown in Figure 3, model performance varies with different parameter settings. For example, in the Attentive FP model on the DM task, the model performed poorly with a large batch size, large number of layers, and low dropout rate. These indicate that increased model complexity might not always lead to better performance, and each model must be tuned appropriately. For the GCN model on the TP task, the model performed better with a smaller batch size, large channel width, and suitable dropout rate. The V shape in the dropout (Figure 3F) implies that a reasonable dropout rate is required to overcome overfitting, while a large dropout rate might lead to insufficient training. In this work, a grid search cross-validation method was used to explore the hyperparameter space. However, it is not possible to explore all possible configurations, and the grid search method does not necessarily lead to the optimum set. A Bayesian optimization approach, as implemented in the hyperopt package [20], may prove useful for future work.

Sample size is an important factor for machine learning models. As reported in this study, graph models reached the best and worst performance in the TP and DM tasks, respectively. This aligns with the comparison of the training size, where the TP task includes 2035 molecules while the DM task has only 753 molecules. The VF and fish tasks have similar sample sizes of 1271 and 956, and thus have comparable performance. Therefore, it is reasonable to conclude that a larger dataset would lead to better performance. In addition, the training datasets in this work lacked a significant number of nontoxic molecules. Real-world applications may not reflect this data distribution, so these models may be limited in such environments and should be used with caution where a large number of molecules have low or zero toxicity. Future research may investigate graph model performance on a dataset curated with regard to real-life scenarios.

In the current study, each graph model was trained independently on four tasks, which does not take advantage of the fact that some tasks share common molecules. Transfer learning should be considered in future applications where a low-level embedding layer can be trained to learn common molecular structures with high-level task-specific layers.

It should be noted that the primary purpose of this study is to evaluate and compare the five selected graph models quantitatively instead of using model interpretation and molecular structural explanation. Attentive FP is highlighted to be unique in producing atomic weights from which a heatmap may be generated for further analysis, but the study is limited in analyzing the structural basis of toxicity.

## 4. Materials and Methods

### 4.1. Data Collection

The dataset was collected from Li et al. [21]. As reported, several publications and databases were utilized to compose a collection of 5048 toxicity data points for chemicals from fish, *Daphnia magna* (DM), *Tetrahymena pyriformis* (TP), and *Vibrio fischeri* (VF). Li et al. [22] was the primary source of data points, with additional toxicity values for TP taken from Ruusmann and Maran [23]. The dataset is freely available at https://github.com/hhaootian/toxicity/blob/main/data/toxicity.xlsx (accessed on 20 May 2023).

The fish toxicity values express median lethal concentration ($LC_{50}$) after 96 h and were taken from data for the fathead minnow (*Pimephales promelas*), guppy (*Poecilia reticulata*), medaka (*Oryzias latipes*), and rainbow trout (*Oncorhynchus mykiss*). Prior work by Li et al., from which this dataset was partially curated, showed strong correlation of toxicity between the four species [22]. The DM data use $LC_{50}$ or half maximal effective concentration ($EC_{50}$) after 48 h. The TP toxicity data express the concentration required for 50% growth inhibition ($IGC_{50}$) after 40 or 48 h. Highly similar data were observed between the two timeframes. The VF values indicate 50% bioluminescence inhibition concentration ($IBC_{50}$) after either 15 or 30 min, with higher preference given to 30-minute data points. The average toxicity value was used for compounds with more than one value for a certain species. For all acute toxicity values, the negative logarithm of the molar concentration (mol/L) was taken.

For each entry, featurization techniques corresponding to each graph model were applied. A molecule is dropped if it fails to produce a proper featurization result. In total, 14 out of 2049 molecules were dropped in the TP task, 4 out of 757 molecules in the DM task, 6 out of 1277 molecules in the VF task, and 9 out of 965 molecules in the fish task. The featurization script to produce cleaned datasets is available at https://github.com/hhaootian/toxicity/blob/main/src/clean.py (accessed on 20 May 2023).

The GAT, GCN, MPNN, and Attentive FP models use MolGraphConvFeaturizer, v2.7.1 which creates graph convolutional representations of molecules. This method encodes the molecular structure, including atoms, bonds, and bond types, directly in the featurization result. This allows for more data-driven machine learning than with the use of traditional molecular fingerprints [24]. The PAGTN model uses PagtnMolGraphFeaturizer, v2.7.1. This also exploits molecular graph convolutions but specifically connects all atoms within the molecule while accounting for interactions between them relative to their distance [15]. These featurizers were implemented in DeepChem [25].

### 4.2. Graph Models

#### 4.2.1. Message-Passing Neural Network

A message-passing neural network (Algorithm 1) is a type of graph neural network that operates on graph-structured data and incorporates message passing, which allows information to be exchanged between nodes in a graph. These nodes represent atoms, while edges between them represent bonds. MPNNs exploit this graph structure to learn interactions between the nodes. The neural network iteratively updates the hidden state of each node by first passing messages and then aggregating information from its neighboring nodes. The MPNN architecture has found success in a variety of applications, including molecular property prediction, due to its ability to process complex molecular graphs [13].

The MPNN architecture comprises three main steps: message passing, feature update among vertices, and readout. First, features are assigned to each atom ($h_{v}$) and bond ($e_{vw}$). Then, nodes send messages to their neighbors, and features are updated accordingly. Finally, a readout function creates graph-level features to be used in machine learning tasks.

(1) $$\begin{array}{cc} m_{v}^{t+1} & =\sum_{w\in N(v)}M_{t}\left(h_{v}^{t},h_{w}^{t},e_{vw}\right) \end{array}$$

(2) $$\begin{array}{cc} h_{v}^{t+1} & =U_{t}\left(h_{v}^{t},m_{v}^{t+1}\right) \end{array}$$

The message-passing protocol consists of two main components: message passing along edges (Equation (1)) and vertex update (Equation (2)). The message function $M_{t}$ aggregates edge features with their incident nodes, while the update function $U_{t}$ updates each node’s hidden state $h_{v}^{t}$ based on the message $m_{v}^{t+1}$ received at iteration t+1. Here, N(v) denotes the neighbors of *v*. In the final readout step, the function *R* combines the atom features to represent the whole graph in a vector $\hat{y}$ (Equation (3)) [14].

(3) $$\begin{array}{c} \hat{y}=R\left(h_{v}^{T}|v\in G\right) \end{array}$$

**Algorithm 1** Message-Passing Neural Network.
 1.Perform multiple rounds of message passing, in which nearby node representations and edge features are combined and node hidden states are updated. 2.For each graph, compute its readout function by combining the representations of all its nodes. 3.Perform the final prediction using an MLP.


#### 4.2.2. Graph Convolution Networks and Graph Attention Networks

Graph convolution networks (GCNs, Algorithm 2) are a form of neural network architecture designed to operate on graph structures using convolutions. They utilize a fixed convolutional operation based on traditional convolutional neural networks (CNNs), either in the spectral or spatial domain of the graph [26]. GCNs typically aggregate feature data from a node’s immediate neighbors to determine its hidden state, which is often used as its final representation. These neural networks can operate directly on molecular graphs, rather than using fingerprint vectors, in order to improve accuracy, efficiency, and interpretability [27].

Graph attention networks (GATs) combine a neural network with an attention mechanism that determines the relative importance of neighboring nodes. Unlike GCNs, which apply fixed convolutional operations, GATs compute attention coefficients for each neighbor and weigh their contributions when updating the hidden state. This hidden state is an aggregation of the weighted contributions of neighboring nodes, providing each node with a tailored representation based on its neighbors. This allows GATs to selectively emphasize different neighboring nodes based on their importance and focus on the most salient aspects of an input, which can also improve the architecture’s interpretability [28].
**Algorithm 2** Graph Convolution/Attention Networks. 1.Update node information using a variant of the GCN/GAT algorithm. 2.For each graph, compute its representation via the following process:
 (a)Apply a gating function to node representations to calculate their weight; then, compute a weighted sum of all representations in the graph. (b)Apply max pooling to the node representations. (c)Concatenate the output of (a) and (b). 3.Perform the final prediction using an MLP.

#### 4.2.3. Path-Augmented Graph Transformer Network

PAGTNs (Algorithm 3) were introduced by Chen et al., in 2019 to solve the problem of GCNs failing to recognize long-range structural relationships in molecules [15]. This type of model utilizes the transformer architecture on graph-structured data. In comparison to other models like GCNs, which aggregate primarily local information on nodes, PAGTNs utilize a globally connected attention framework. This means that each node attends to all other nodes while still focusing on the most important ones. PAGTNs exploit path features to learn long-range relationships between nodes without needing several layers. These paths may provide valuable information on molecular substructures, which could explain the success of PAGTNs in molecular property prediction.
**Algorithm 3** Path-Augmented Graph Transformer Network. 1.Update node information in graphs using a linear additive attention mechanism. Attention weights are determined by concatenating the node and edge features for each bond. 2.Complete several rounds of message passing and updating node information. 3.Each layer has residual connections to the previous layer. 4.Compute the final molecular representation by aggregating the representations of all nodes. 5.Perform the final prediction using a linear layer.

#### 4.2.4. Attentive FP

The Attentive FP model (Algorithm 4) is a GNN architecture that represents molecules using molecular fingerprints and a graph attention mechanism. Rather than calculating molecular conformation or connecting all atoms with virtual edges, as in the MPNN model, Attentive FP prioritizes interactions between nearby atoms while still considering the effects of distant ones, beyond a node’s immediate neighbors. This is made possible by the attention mechanism, which assigns greater weights to more important substructures in order to focus on the most relevant parts of a molecule. This property of Attentive FP can be leveraged to interpret the model, which is key for comparing it to existing domain knowledge and potentially gaining insight into novel chemical patterns [5].
**Algorithm 4** Attentive FP. 1.Initialize node information by combining node and edge features, along with a round of message passing. 2.Update node representations via several rounds of message passing. 3.Compute the overall representation of each graph by aggregating all node representations and applying a gated recurrent unit (GRU). 4.Perform the final prediction with a linear layer.

Graph models were implemented in Python (v3.8.11) DeepChem [25] (v2.7.1), PyTorch (v2.0.0), and DGL (v1.1.0) packages.

### 4.3. Performance Metrics

Three metrics were calculated to quantify model performance on each regression task. With *n* pairs of true value $Y_{i}$ and predicted value ${\hat{Y}}_{i}$, mean square error (MSE), mean absolute percent error (MAPE), and R-squared (R^2^) are calculated as follows:

(4) $$\begin{array}{cc} \mathrm{MSE} & =\frac{1}{n}\sum_{i=1}^{n}{\left(Y_{i}-{\hat{Y}}_{i}\right)}^{2} \end{array}$$

(5) $$\begin{array}{cc} \mathrm{MAPE} & =\frac{1}{n}\sum_{i=1}^{n}\left|\frac{Y_{i}-{\hat{Y}}_{i}}{Y_{i}}\right| \end{array}$$

(6) $$\begin{array}{cc} R^{2} & =1-\frac{\sum {\left(Y_{i}-{\hat{Y}}_{i}\right)}^{2}}{\sum {\left(Y_{i}-\bar{Y}\right)}^{2}} \end{array}$$

## 5. Conclusions

The development of graph models provides an alternative method to QSTR models for acute toxicity prediction. In this study, a pipeline was provided to compare different graph models for this purpose. Performance metrics were evaluated and key differences were reported among five popular graph models, namely, MPNNs, GCNs, GATs, PAGTNs, and Attentive FP. As reported, the Attentive FP model performed the best with a low prediction error, a high coefficient of determination R^2^, and good interpretability. The importance of parameter fine-tuning and sample size was discussed, along with the potential application of transfer learning.

## Figures and Tables

**Table 1:** Parameters being fine-tuned in graph models.

	MPNN	GCN	GAT	PAGTN	Attentive FP
Batch size	[32, 128]				
Dropout	[0, 0.1, 0.2]				
Number of GNN layers	N/A	N/A	N/A	[1, 2, 4]	
Number of graph convolution layers	N/A	[64, 64], [128, 128], [256, 256]		N/A	N/A
Number of units	[64, 128, 256]	N/A	N/A	N/A	N/A
Number of atom output features	[15, 30, 60]	N/A	N/A	N/A	N/A
Size for the hidden node features	N/A	N/A	N/A	[64, 128, 256]	N/A

N/A means this parameter is not available in the tunable model parameters.

**Table 2:** Model performance evaluated on test set.

Task	Model (Rank)	MSE	MAPE	R^2^
DM	MPNN (5)	1.223 ± 0.115	0.256 ± 0.010	0.409 ± 0.055
	GCN (3)	0.942 ± 0.074	0.234 ± 0.013	0.545 ± 0.036
	GAT (2)	0.937 ± 0.031	0.232 ± 0.006	0.548 ± 0.015
	PAGTN (4)	1.060 ± 0.102	0.247 ± 0.019	0.489 ± 0.049
	Attentive FP (1)	0.903 ± 0.041	0.209 ± 0.011	0.564 ± 0.020
VF	MPNN (4)	0.759 ± 0.068	0.227 ± 0.007	0.455 ± 0.049
	GCN (2)	0.709 ± 0.025	0.219 ± 0.011	0.491 ± 0.018
	GAT (5)	0.824 ± 0.113	0.223 ± 0.013	0.408 ± 0.081
	PAGTN (3)	0.729 ± 0.032	0.211 ± 0.008	0.476 ± 0.023
	Attentive FP (1)	0.622 ± 0.064	0.195 ± 0.008	0.553 ± 0.046
TP	MPNN (4)	0.246 ± 0.013	0.119 ± 0.003	0.778 ± 0.012
	GCN (2)	0.211 ± 0.013	0.110 ± 0.003	0.810 ± 0.011
	GAT (3)	0.226 ± 0.018	0.116 ± 0.003	0.796 ± 0.016
	PAGTN (5)	0.254 ± 0.014	0.117 ± 0.005	0.771 ± 0.013
	Attentive FP (1)	0.183 ± 0.015	0.102 ± 0.003	0.835 ± 0.013
Fish	MPNN (5)	0.865 ± 0.098	0.213 ± 0.030	0.529 ± 0.053
	GCN (2)	0.646 ± 0.037	0.203 ± 0.010	0.644 ± 0.020
	GAT (4)	0.749 ± 0.049	0.234 ± 0.005	0.592 ± 0.027
	PAGTN (3)	0.669 ± 0.043	0.183 ± 0.014	0.636 ± 0.023
	Attentive FP (1)	0.623 ± 0.037	0.170 ± 0.012	0.666 ± 0.020

## Data Availability

The data and codes to reproduce the results are available at https://github.com/hhaootian/toxicity (accessed on 20 May 2023).

## Abbreviations

The following abbreviations are used in this manuscript:

AOT	Acute oral toxicity
ADMET	Absorption, Distribution, Metabolism, Excretion, and Toxicity
CNN	Convolutional neural network
DM	*Daphnia magna*
$EC_{50}$	Half maximal effective concentration
GAT	Graph attention network
GCN	Graph convolution network
GRU	Gated recurrent unit
$IBC_{50}$	50% bioluminescence inhibition concentration
$IGC_{50}$	50% growth inhibition concentration
$LC_{50}$	Median lethal concentration
MAPE	Mean absolute percent error
MLP	Multilayer perceptron
MPNN	Message-passing neural network
MSE	Mean squared error
PAGTN	Path-augmented graph transformer network
QSTR	Quantitative structure–toxicity relationship
TP	*Tetrahymena pyriformis*
VF	*Vibrio fischeri*
